# Genome Expression Pathway Analysis Tool – Analysis and visualization of microarray gene expression data under genomic, proteomic and metabolic context

**DOI:** 10.1186/1471-2105-8-179

**Published:** 2007-06-02

**Authors:** Markus Weniger, Julia C Engelmann, Jörg Schultz

**Affiliations:** 1 Department of Bioinformatics, Biocenter, University of Würzburg, 97074 Würzburg, Germany

## Abstract

**Background:**

Regulation of gene expression is relevant to many areas of biology and medicine, in the study of treatments, diseases, and developmental stages. Microarrays can be used to measure the expression level of thousands of mRNAs at the same time, allowing insight into or comparison of different cellular conditions. The data derived out of microarray experiments is highly dimensional and often noisy, and interpretation of the results can get intricate. Although programs for the statistical analysis of microarray data exist, most of them lack an integration of analysis results and biological interpretation.

**Results:**

We have developed GEPAT, Genome Expression Pathway Analysis Tool, offering an analysis of gene expression data under genomic, proteomic and metabolic context. We provide an integration of statistical methods for data import and data analysis together with a biological interpretation for subsets of probes or single probes on the chip. GEPAT imports various types of oligonucleotide and cDNA array data formats. Different normalization methods can be applied to the data, afterwards data annotation is performed. After import, GEPAT offers various statistical data analysis methods, as hierarchical, k-means and PCA clustering, a linear model based t-test or chromosomal profile comparison. The results of the analysis can be interpreted by enrichment of biological terms, pathway analysis or interaction networks. Different biological databases are included, to give various information for each probe on the chip. GEPAT offers no linear work flow, but allows the usage of any subset of probes and samples as a start for a new data analysis. GEPAT relies on established data analysis packages, offers a modular approach for an easy extension, and can be run on a computer grid to allow a large number of users. It is freely available under the LGPL open source license for academic and commercial users at .

**Conclusion:**

GEPAT is a modular, scalable and professional-grade software integrating analysis and interpretation of microarray gene expression data. An installation available for academic users can be found at .

## Background

### Introduction

Gene expression analysis using microarrays opened new insights into the living cell, revolutionizing biological research in many fields. Gene expression of a whole system can be measured at once, yielding information about the mRNA level of every gene. Microarrays have become a standard tool for gene expression measurement in biology and medicine. Their application ranges from identification of gene expression changes in different states of the cell cycle over the classification of disease types to drug development. Although microarrays are widely used, a fundamental challenge is to cope with the immense amount of data generated. Therefore special software packages have been developed, capable of handling the analysis of microarray data. Still, we think that many of the existing tools are not optimal in respect of usability and integration. To date, most freely-available programs split the data analysis into two parts: In the first, statistical methods are used to identify lists of 'interesting' genes, in the second these lists are searched for biological relevance. Although these two steps are dependent on each other and should be highly interconnected, currently most analysis tools lack an integration of these steps. In the following, we will give an overview of selected tools.

### Existing Tools

One of the most sophisticated software for microarray data analysis is the Bioconductor toolkit [1], based on the R statistical programming language [2]. Most algorithms developed for microarray data analysis are available within this package. Unfortunately, Bioconductor is a text-driven command line tool and does not provide an easy-to-use graphical interface. Therefore, it offers advanced analysis methods and the possibility of easy extension only for professional users, and is difficult to use for people unskilled in R. Results could be misinterpreted if people are not understanding the data they are working with or the way to perform the analysis. To solve this problem, different tools were developed wrapping the Bioconductor toolkit for an easier usage. *AMDA *[3] is an R package, providing a graphical user interface and a workflow for the analysis of Affymetrix microarray data. *CARMAWeb *[4] acts as a web-based user interface, making the Bioconductor modules available for data analysis over the internet.

Besides Bioconductor, other data analysis tools are available. *Expression Profiler *[5] offers an integrated, web based approach for microarray data analysis. Various normalization, filtering, between-group-testing, clustering, cluster comparison and GO term enrichment analysis methods are available. Expression Profiler integrates analysis methods in an application-like web interface. *GEPAS *[6] is also a widely used web-based approach for microarray data analysis. In addition to the functionality of Expression Profiler, it also offers class prediction methods, survival analysis and multiple tree viewers. GEPAS functionality is split up into a number of tools, connected by the same file format. The user interface is more web-styled than Expression Profiler, making the usage more complicated for untrained users.

Other Tools are not web-based, but installed on the local machine. *EXPANDER *[7] includes biclustering methods and analysis methods regarding regulatory elements. *TM4 *[8] is a collection of 4 programs, covering all computational steps for microarray analysis. *TM4 *includes spot detection/image analysis, data normalization and data analysis, linked together by the same file format. The data analysis part includes, beside other analysis methods, support vector machines, gene shaving and relevance networks.

All these programs share the focus on the data analysis part, but most of them lack tools for the interpretation of the results. Only *GEPAS *offers with *Babelomics *[9] an approach into data interpretation. On the other hand there exist tools focusing on the interpretation of analysis results. Besides many others, *WebGestalt *[10] offers biological term enrichment analysis, protein domain tables, tissue expression analysis, links to chromosome location and textmining analysis. The widely used *DAVID *[11] allows an enrichment analysis for GO categories, pathway enzymes, protein domains and other biological terms. *Cytoscape *[12] supports the integration of network information with microarray gene expression data. Other tools for acquiring gene set information are *MAPPFinder *[13], *GFINDer *[14] and *Pathway Explorer *[15]. The *Ensembl *[16] annotation system *ENSMART *allows the user to perform a genome information search and retrieval for sets of genes, but does not help in exploring the information associated with the gene set. All these tools provide annotation ability, the drawback of these tools is the inability of an integrated analysis. They require precalculated gene sets as input, needing other tools for normalization, clustering and subset determination.

### GEPAT

For interpreting microarray analysis results with the tools described above, researchers need first to obtain a list of differential expressed genes from an analysis program, and use this list in an interpretation program to get biological information for the results. This might prove feasible for smaller number of experiments, but is time-consuming and complicated if used for larger numbers.

As we were unhappy with the separation of analysis and interpretation, we developed our own tool, GEPAT. GEPAT offers combined genome-, expression- and pathway analysis and interpretation methods. Our idea was the integration of gene expression data evaluation with the cellular regulation and interaction network. Therefore, we provide gene annotation for the probes on the microarray and allow the visualization of analysis results on metabolic pathways and gene interaction networks. GEPAT includes different biological databases, making them directly usable in data analysis and interpretation. As a large number of databases require lots of disk space and the analysis methods demand much computation power, we developed GEPAT as a web-based toolkit. GEPAT offers an application-like user interface with menu bar and dialog boxes for easy usage. The installation as server system allows either installation and usage on a single computer, installation on a web server for use within a workgroup, or installation on a web server connected to a computer cluster for large user groups. GEPAT is distributed under LGPL and can be freely downloaded [17], an installation on our server can be used by academic users [18]. For an easy start, GEPAT provides a video tutorial for the basic steps, and offers online help for most functions. For a first impression of GEPAT, a guest login is available, preloaded with microarray data from cancer type classification [19] and cancer subgroup profiling of diffuse large B-cell lymphoma [20], including chromosomal alteration information [21]. All figures in this paper are based on the B-cell lymphoma dataset.

## Implementation

### Web Server

*GEPAT *is implemented in the Java programming language [22] and requires a J2EE-compatible servlet container to run. Our server installation uses Apache Tomcat [23] as base. The JavaServer Faces technology is used for the generation of web pages. This technology offers a Model-View-Controller-based programming approach for internet applications, allowing application development similar to desktop applications. Access control and image generation are implemented using Java Servlets. All databases used and algorithms implemented in GEPAT are wrapped in modules. The program itself provides only user management and data management capabilities, all other functionality is modularly implemented. This allows an easy extension with new databases or new analysis methods. Modules are used for import of gene expression data, subset selection of probes or samples, gene information, analysis and interpretation methods. The currently implemented modules for data analysis can either run calculation on the server itself or calculation can be directed to a computer grid running a DRMAA-compatible grid engine [24]. In our case, the computation scripts are run on our 10-node cluster system, based on the Sun Grid Engine [25]. For data analysis, we used the powerful abilities of the Bioconductor toolkit combined with an easy-to-use interface. For graph layout and visualization, the JUNG [26] graph library is used.

### Databases

The modular approach of GEPAT allows the usage of any database by developing new modules. We have already integrated modules for the access of some important biological databases as Ensembl [16]. As the format of most databases was not suitable for our purposes, we reformatted these databases for our needs. For storage a mySQL 5 [27] database server is used. GEPAT provides scripts for the creation of the database tables and the conversion of already existing databases into these tables.

For gene annotation, we found no available database for all clone identifier mapping purposes needed. Therefore we created our own database. We used the UniGene database (Build #197, 12/2006) [28] to provide a mapping from cDNA Clone identifiers (ids) and Genbank ids to UniGene clusters, and used the UniGene information for Ensembl gene entries to map each probe to an Ensembl gene ID. Affymetrix probe identifiers are directly annotated with the information provided from the Ensembl database (41_36c). At the moment, our database is focused on human datasets, support for other organisms will follow in the future.

Unluckily, Ensembl-identifiers do not exist for all probes, as some probes are derived from EST tags for which no gene is annotated, or some probes may bind to more than one mRNA. If an Ensembl identifier is available for a probe, the Ensembl database entry is used to gain information about gene name, chromosomal location, proteins, GO Annotation and enzymatic activity. All data annotation in GEPAT is performed via the Ensembl identifier. The identifier used for annotation is selected automatically out of the array files, or can be selected by the user for tabular file input.

Linking a probe to a gene is necessary for interpretation of the results, but may lead to various problems. Microarray probes may not only hybridize with one specific mRNA, but crosshybridize with mRNAs of different genes. It is also possible that a probe detects only one specific splice variant of a gene, while another probe detects all splice variants. Different probes may hybridize more or less efficiently with the mRNA they were designed for. And at last, it is not always sure if the probes contain the cDNA-material they are supposed to. Therefore it is necessary to compare the sequence of somehow interesting probes with a sequence database, to make sure annotation was right, and to verify the results of the microarray analysis by other experimental methods.

## Results

Microarray experiments generate a large amount of data in a very short time. In most cases it is not desirable to work with all these data at once. Only few probes may be differentially expressed, so in some cases it is useful to limit data interpretation to only these probes. The array dataset may consist of numerous subsets of somehow different samples. For the probe and sample set, subsets may be used to focus only on a specific group, or to compare two groups. Defining and working with different subsets for any kind of analysis and interpretation is one of the main concepts of GEPAT. For visualization purposes, a working set can be defined, and all output is generated for this working set. For example, as it is not always desirable to have all data mapped to a metabolic pathway map, by limiting the working set to a subset of all probes, the amount of displayed probes shown on a pathway map can be limited.

The subsets used in GEPAT can be selected by different characteristics. For an easy access in analysis, a subset can be named and stored as "group". For example, in a clinical study, all samples belonging to a specific type of disease may be stored in a group with the disease name. This allows quick data analysis by just selecting the desired disease groups. As source for the selection of subsets, either the whole dataset or subsets defined as a group can be used. It is also possible to use a previous subset as source for the selection, allowing to subset subsets. An overview of possible criteria for subset selection is given in Table 1. As an example, it is possible to select all differentially expressed genes, to limit this set to all genes located in the nucleus, and to limit this set further to all genes that originate from a specific chromosome. Any other combination of subset selection criteria is possible. The probe and sample subset selection process is handled modular, allowing an easy extension with yet unimplemented selection modules for other criteria.

**Table 1:** Possible criteria for selection of probe and sample subsets

Probe set	Sample set
Name Search	Name Search
Groups	Groups
GO Category	k-Means-Cluster Analysis Results
k-means-Cluster Analysis Results	Principal Component Analysis Results
t-test Results	
KEGG Maps	
Chromosomal location	

GEPAT includes the following processing steps for microarray data:

• Import and normalization of microarray gene expression data

• Information for specific genes in the dataset

• Various analysis methods for microarray data, including a moderate t-test and clustering

• Interpretation methods for subsets of the data

The analysis and interpretation steps can be performed in any order, allowing the usage of interpretation results as a start for further analysis. The following text describes the processing steps in detail.

### Data Import

#### Data Input

Data input is an important step in data analysis. Most existing programs require processed data in a specific format, frequently tab-separated tables, or support only a limited amount of formats. To allow broad usage of different input file formats, we decided to use a modular system allowing the extension for any type of file format. All input files are handled by a specific module, and following the import the data is stored in an internal, format-independent and fast-accessible format on the server.

At the moment, three different modules are available for data import. The first module enables data import for tab-separated data files, containing either already normalized data or unnormalized single- or dual-channel data. The other two modules allow the import of oligonucleotide and cDNA microarray data. Affymetrix oligonucleotide arrays are handled by read.affybatch, the cDNA-import uses read.maimages R methods. All formats supported at the moment are listed in Table 2.

**Table 2:** Supported microarray input file formats

**Oligonucleotide**
Affymetrix CEL Files (Human)
**cDNA**
Agilent Feature Extraction
ArrayVision
BlueFuse
GenePix
ImaGene
QuantArray
SPOT
Stanford Microarray Database

**Tabular**
Unnormalized Dual-Channel Data
Unnormalized Single-Channel Data
Normalized Data

**Figure 1:**
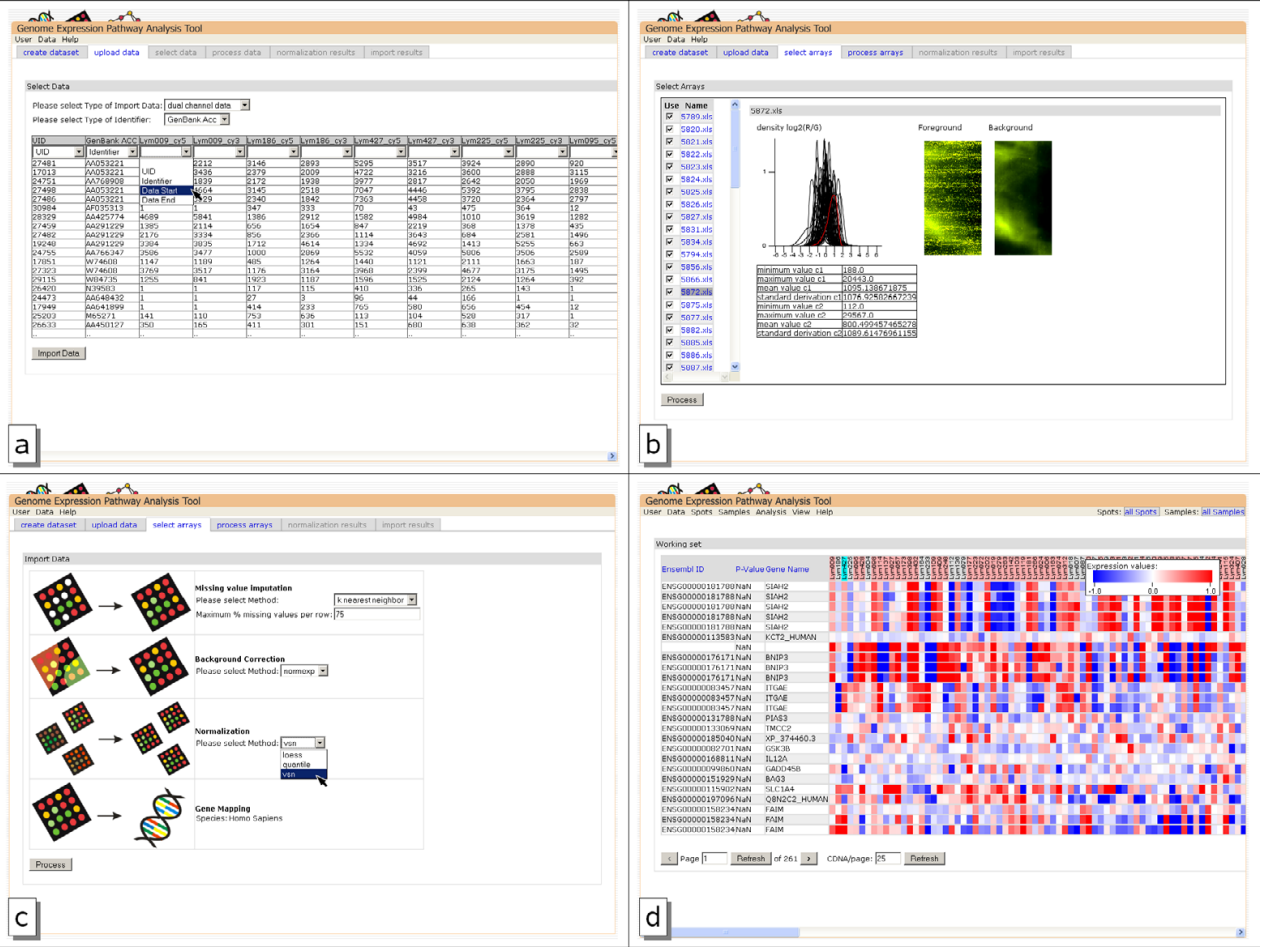
**Data import pages**. a)import of tabular dual channel unnormalized data. Below the heading of the columns drop-down boxes are used to provide information for import. b)Microarray import view. The table on the left side can be used to select microarrays by name, the right side shows the scanned microarray image and data characteristics. The value distribution of all arrays is given in black, the selected array is marked in red. c)Normalization parameter selection page d)overview of imported and annotated microarray expression data. Probes are shown in the rows, the columns show gene information and sample expression.

For saving bandwidth and mouseclicks, multiple array files are imported wrapped in a Zip-File. This allows the upload of a large amount of arrays without separate uploading of each single file. Upload of tab-separated microarray files provides an easy selection of identifier and data columns, shown in figure 1a. After upload, the data channels of the arrays and the data characteristics can be inspected visually to allow a quick identification of blurred or otherwise erroneous arrays. The microarray selection process is shown in figure 1b. Here arrays can be skipped, removing them from further processing. After the selection of microarrays, data normalization methods can be applied to the data.

#### Normalization

Normalization of microarray data is needed to remove variations in gene expression levels caused by the measurement process, enabling the comparison of different microarrays with each other. It aims to remove the systematic effects while keeping the most of the signal, and brings the data from different microarrays onto a common scale.

Before normalization, missing value imputation can be performed to fill outmasked probes with the k nearest neighbors averaging method provided by the impute package [29] of Bioconductor. Missing value imputation offers an established method to compute values for flagged probes. This allows the usage of analysis methods not capable of handling unknown data values, but may lead to false results, as imputed values may not reflect the real gene expression levels.

After missing value imputation, a normalization method must be chosen. Most normalization methods distinguish between within- and between-array normalization. Within-array normalization normalizes the expression log-ratios of two-color spotted microarray experiments so that the log-ratios average to zero within each array or sub-array. Between-array normalization normalizes expression intensities so that the intensities or log-ratios have similar distributions across a series of arrays. Figure 1c shows the normalization configuration page of GEPAT.

GEPAT uses the package *limma *[30] for normalization of two-color microarrays. Different methods are available: One method combines loess within-array normalization [31] and scale between array normalization. The loess method fits the arrays to a polynomial surface, the scale-method scales the log-ratios to have the same median-absolute-deviation across arrays. The other methods use quantile [32] to ensure that the intensities have the same empirical distribution across arrays and across channels or vsn [33] for a robust estimation of variance-stabilizing and calibrating transformations for microarray data. Background correction can be performed via the normexp-method. This method results in a smooth monotonic transformation of the background subtracted intensities such that all the corrected intensities are positive.

For the normalization of Affymetrix arrays the expresso-function of the affy-package is used. Perfect match adjustment ensures that only perfect match oligonucleotides are used for further calculation. For the calculation of the expression values, medianpolish is used. No background-correction is performed. As normalization methods loess, quantile and vsn can be chosen. After normalization, annotation is performed, and data is ready for further analysis. After import, the dataset is shown in an overview table, giving annotation information for the spots and showing the gene expression values for the samples. Figure 1d shows an overview table of the B-cell lymphoma test dataset.

### Gene Information

To gain insight about the biological function of the genes on the microarry chip, different sources can be used for gene information. We include some of the most important sources in GEPAT. Gene information is available in most analysis and interpretation views. A click on a probe or gene opens a new window, giving all available gene information. A tab-bar at the top of the page can be used for changing between the different types of information. Gene Information is also modularized and therefore easily expandable with additional information.

#### Gene Information

**Figure 2:**
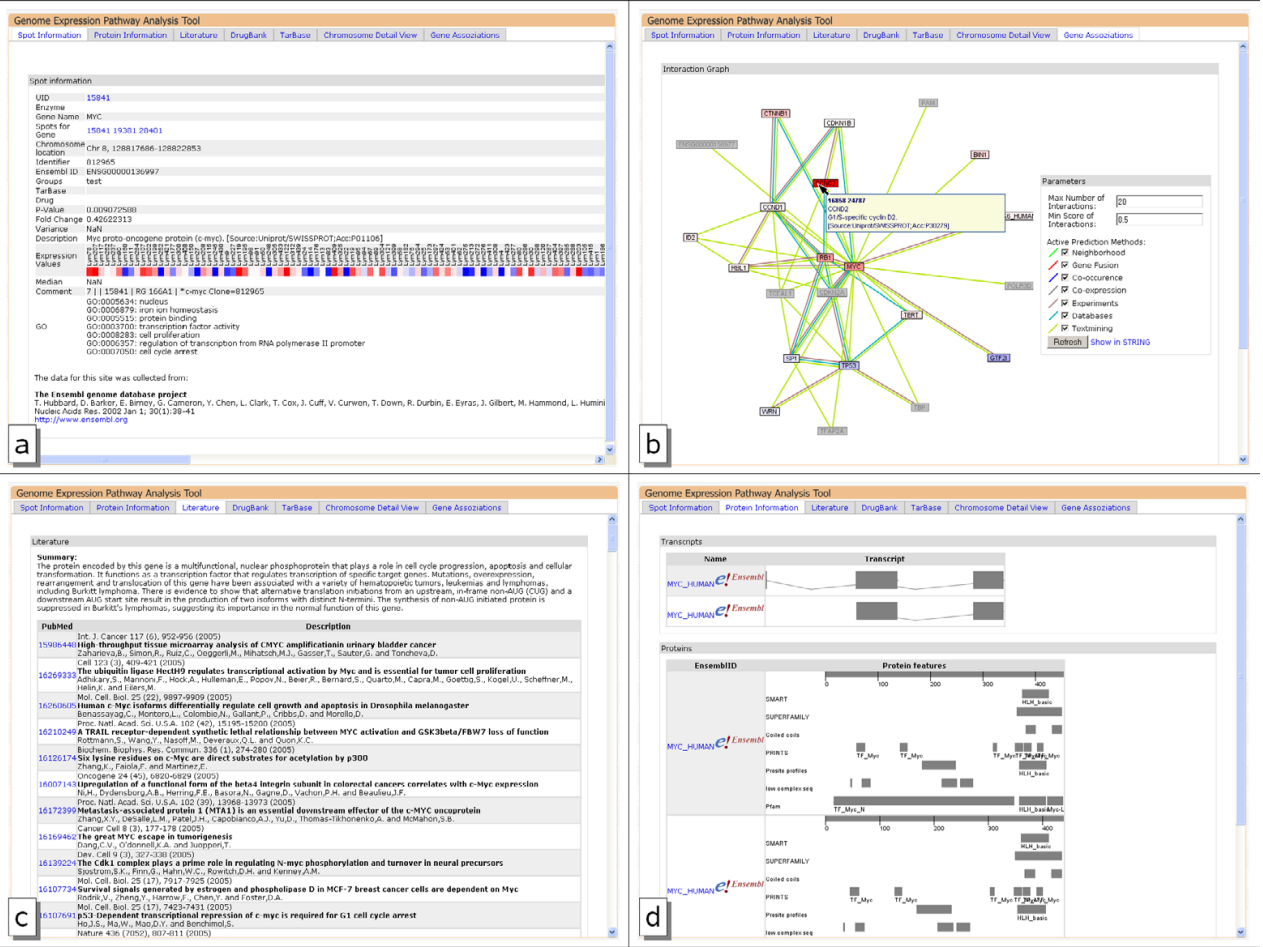
**Gene information pages**. a)Shows an overview of probe 15841 that measures expression level of the *MYC *gene. The information shown can be modularly extended. b)associated genes for this probe, overlaid with differential gene expression results c)shows literature references and a short description derived from RefSeq, d)shows protein information for the gene. The upper part shows the coding regions, the lower part shows features for the different transcripts.

For each gene in GEPAT a quick overview showing gene information can be accessed. We offer a subset of the Ensembl gene information, and link to the corresponding Ensembl page for further information. Besides gene name a short description of gene function, chromosomal location, expression values, GO identifier and enzymatic activity are shown if available and link directly to the corresponding pages in GEPAT. An example of an Ensembl information page for the *MYC *gene is given in figure 2a. The information given on the dataset overview page is a subset of the information given on the gene information page, and is modularly expandable.

#### Gene Associations

The STRING database [34] provides an overview of the physical and functional associations and interactions between proteins. STRING integrates known and predicted protein interaction data from a variety of sources. These associations can be shown in a summary network, displaying the genes as nodes, and different kinds of associations as edges. In GEPAT, we adopted this kind of view. A local instance of the STRING database can be used with GEPAT, and we provide a mapping from Ensembl genes to STRING proteins.

To give an overview of genes interacting with the selected gene, a graph view displaying associated genes can be generated. Similar to the STRING database, possible gene associations are gene neighborhood, gene fusion, co-occurrence, co-expression, experiment, databases and text mining. To keep the graph understandable, the maximal count of nodes can be limited by score and number. For an easier interpretation of the data, differential expression results can be overlaid. A mouse click on a node selects the new gene as center of the graph, allowing browsing through the gene interaction network. The gene association graph for *MYC *is shown in figure 2b.

#### Literature References

Literature about genes can be found in various journals. To give a quick overview of scientific articles related to a gene, we implemented a literature reference view. We used the RefSeq [35] database from NCBI and the Ensembl RefSeq annotation for the genes to find literature references. For each reference, journal, author and title are provided, and a pubmed outlink offers quick access to abstract and full text. If available in RefSeq, a short summary of the gene function is given, as shown in figure 2c for the *MYC *gene.

#### Protein Information

Although microarrays designed for resolving different splice variants of genes [36] are starting to get available, most actual microarray techniques provide information on gene level. Nevertheless, sometimes it is necessary to gain information about the proteins derived from these genes. This information is provided in the protein information table. The protein information is drawn out of the Ensembl database, a direct link to the Ensembl website is provided for each protein. The protein information table shows the different possible transcripts of a gene, and provides information about the features, e.g. protein domains, of each protein build out of these transcripts. Figure 2d shows the protein information page for *MYC*.

### Data Analysis

A wide variety of data analysis methods is available for gene expression data. We decided to implement differential expression analysis, clustering methods and an analysis of chromosomal alterations in GEPAT. As all analysis methods are implemented as modules, new analysis methods can be added quite easily. With our subset-selection procedure, it is possible to take any probe or sample subset as input for the data analysis methods. The results of the analysis can again be used as criteria for subset selection.

#### Differential Expression

An important analysis of microarray data is the comparison of expression profiles from different sample groups. Different kinds of tests are available; one of the most advanced is the moderate t-statistics, as it provides stable results even for experiments with small numbers of arrays. We use the limma package of Bioconductor for this analysis [30]. Two sample subsets can be specified and compared. For each probe, the log_2 _fold change and p-value are calculated. Benjamini-Hochberg and Benjamini-Yekutieli multiple testing adjustment methods can be applied on raw p-values. These multiple testing correction methods control the false discovery rate, the expected proportion of false discoveries amongst the rejected hypotheses. The false discovery rate is a less stringent condition than the family wise error rate, so these methods are more powerful compared to other methods, e. g. the Bonferroni correction.

**Figure 3:**
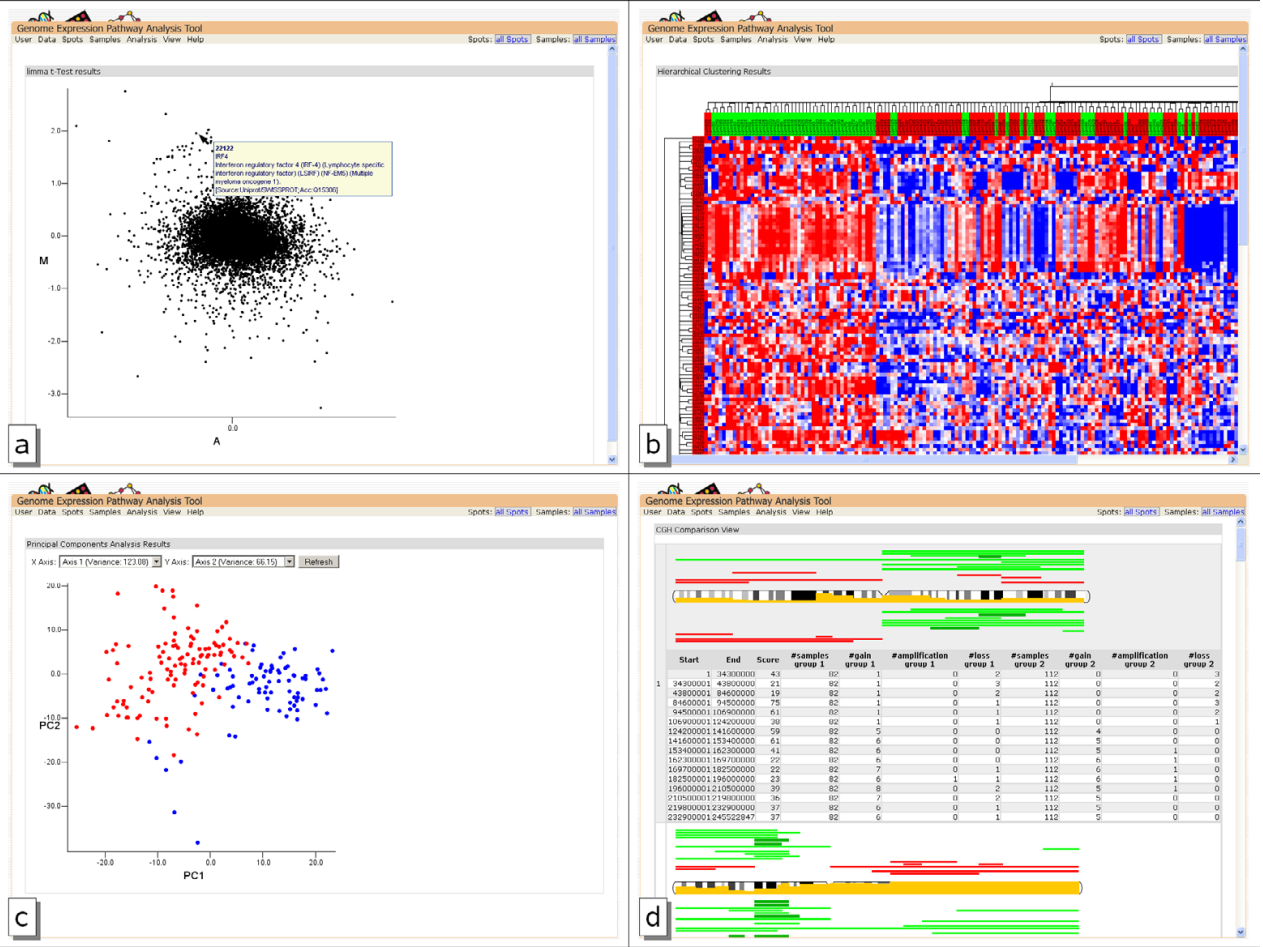
**Data Analysis result views**. Results are shown for activated B-cell (ABC) type cancer samples and germinal center B-cell (GCB) type cancer samples: a)M/A plot of moderate t-test result comparing ABC with GCB b)hierarchical clustering results. The color of the samples marks the different disease types. c)PCA analysis results. Characteristical probes for disease were used as source for clustering. d)CGH profile comparison. The yellow bar in the chromosome shows the difference between the profiles. Above the chromosome the CGH Profile of the ABC group is shown, the GCB group is shown below.

The results can be visualized in an M/A-Plot, allowing an overview of the data distribution. The Y Axis shows the M value, the log_2_-fold change of probe values in the different groups. The X Axis shows the A value, the average expression level for the probe across all the arrays and channels. Additional information is provided via mouse cursor tooltips; a click on a spot provides full information for a probe. An example of an M/A plot is given in figure 3a.

The fold change of differential expression of the compared groups can be mapped onto the visualization components on GEPAT. This allows a direct view of the differential expression on pathways or interaction networks. An important aspect of the t-test is its usage in testing a hypothesis, as it provides error probability values for each tested probe.

#### Clustering

Clustering means the partitioning of data into subsets (clusters), so that each element of the subset shares a common feature. Clustering methods allow visual insight into the data and can be used for class discovery, e.g. for finding disease categories among experiment samples. GEPAT offers the widely used hierarchical clustering, principal component analysis (PCA) and k-means clustering as unsupervised clustering methods.

The hierarchical clustering method is based on the dist and hclust commands of R. Clustering methods include the widely used unweighted pair-group method using arithmetic averages (UPGMA), single linkage, complete linkage and Ward's algorithm. The single linkage method, closely related to the minimal spanning tree, adopts a 'friends of friends' clustering strategy, the complete linkage methods finds similar clusters, whereas Ward's minimum variance method aims at finding compact, spherical clusters. Figure 3b shows an example of hierarchical clustering results.

PCA is a technique for retrieving information out of a dataset by dimensionality reduction, retaining those characteristics of the dataset that contribute most to its variance. GEPAT can perform PCA on the sample data of the dataset. The samples are shown in a two-dimensional plot, where the principal components for each direction can be chosen freely. A lasso-like selection function provides easy subset selection based on the clustering results. The results of PCA clustering are shown in Figure 3c.

The k-means clustering requires a user input, the expected number k of clusters. GEPAT uses the kmeans command of R to perform a clustering based on the Hartigan-Wong algorithm. As a result, k clusters are returned, and can be used in subset selection for further analysis. These subsets can even be used as base for further clustering, allowing the analysis of complex datasets step by step.

#### Value Calculation

Other characteristics of the microarray data can be calculated using the expression values. Median and variance can be calculated for all probes and samples, or only for specific subsets of the data. This allows using probes with the highest variance across samples for further analysis.

#### CGH Data Analysis

GEPAT not only handles microarray data, but is also able to handle additional information for each sample. In cancer datasets, most samples not only differ by gene expression, but have a specific profile of genetic alterations. Comparative genomic hybridization (CGH) is a well-established method that allows the detection of chromosomal imbalances in entire genomes. This technique is widely used in routine molecular diagnostics [37], and many experiments combine CGH and microarray data. We developed a data analysis module capable of comparing the CGH-profile of two sample groups. An unpaired Wilcoxon-Rank test is performed on each chromosomal segment, for comparison of both sample groups. The resulting p-value is plotted directly on the chromosome view, along with the CGH profiles of every group, allowing a quick identification of differing parts. Figure 3d shows a CGH profile comparison example for the lymphoma test dataset.

### Data Interpretation

While performing the analysis steps on the data, sets of interesting genes will be found. Methods for correlating these data with prior biological knowledge are necessary. We developed different modules to facilitate the interpretation of these genes and gene sets in a cellular context. The modules are fully integrated with the analysis steps described above and with each other. Therefore, an interpretation can be performed on any subset of data. This integration is a major focus of GEPAT and distinguishes it from many other available tools for the analysis of gene expression data. Data Interpretation in GEPAT is modularly extensible, allowing implementation of any yet unimplemented interpretation method. Out of each Data Interpretation view, gene information can be provided for each probe.

#### GO Term Enrichment Analysis

At the moment, an automatic ontological approach is one of the most popular methods to gain insights into a set of differentially expressed genes. The Gene Ontology project [38] provides a set of structured vocabularies to describe molecular function, biological process, and cellular component in a hierarchical manner. For interpretation of the data, the GO profile of a subset of genes is compared to the GO profile of a reference set, in most cases all genes of the microarray. The change in the relative frequency of GO terms is used to measure enrichment of GO terms in the subset. A large number of tools exists for performing these analysis for a given list of genes [39]. Out of the different statistical tests used by these tools, we chose an analysis based on a hypergeometrical distribution for GEPAT, as it is an appropriate model for the probability that a certain category occurs x times just by chance in the list of differentially expressed genes. Because of the directed acyclic graph structure of GO multiple testing correction for GO term enrichment analysis is not easy to perform and is still discussed [40], and therefore not provided at the moment.

**Figure 4:**
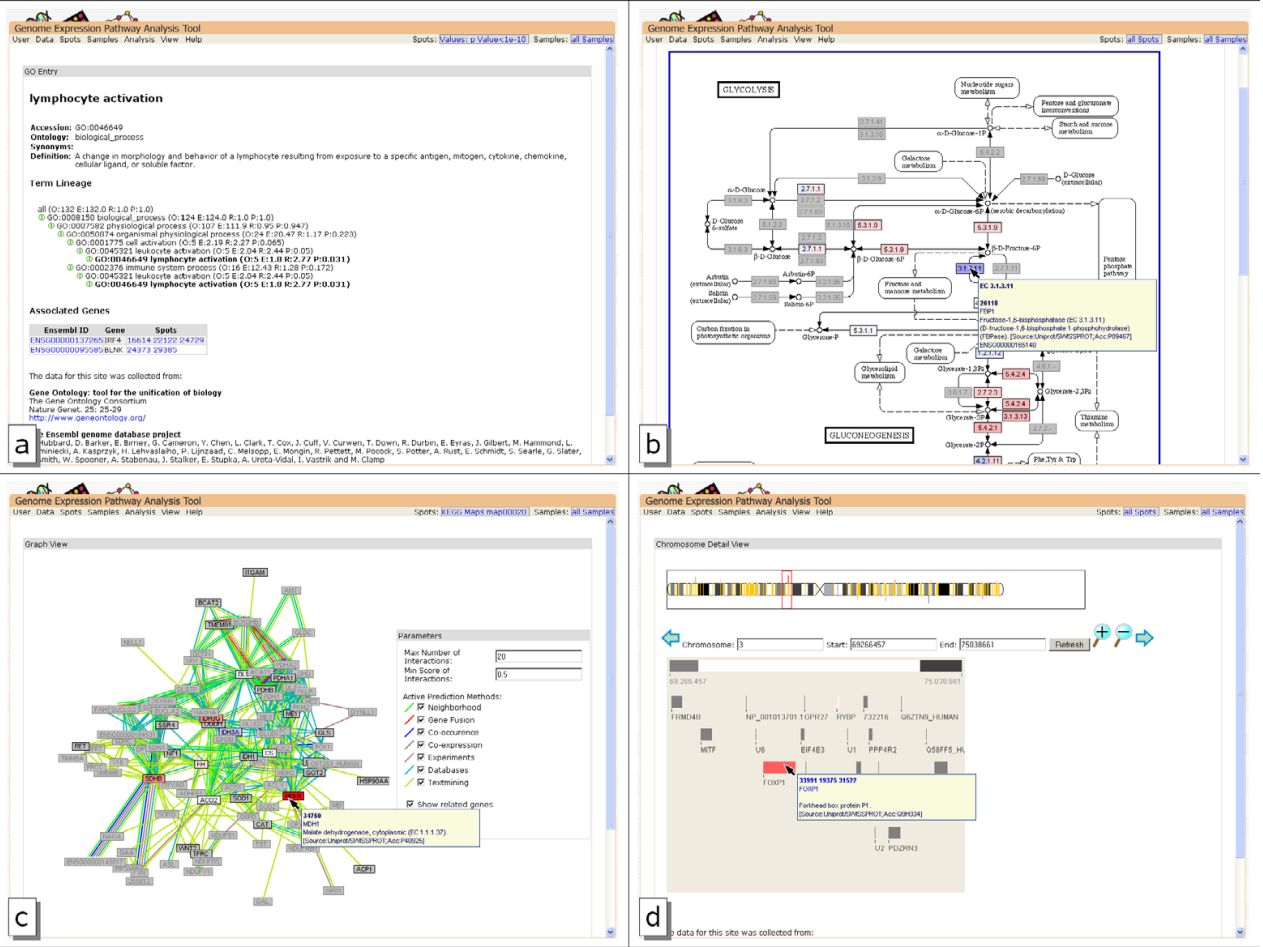
**Views of data interpretation**. The overlaid differential expression values are the result of the t-test shown in figure 3a. Node colors reflect the differential gene expression. The light gray nodes represent associated genes not on the array. a)Comparing the genes with the lowest p-value shows enriched lymphocyte activation in the GO Term enrichment analysis. b)Glucolysis KEGG map overlaid with differential expression result. c) gene association network of the glucolysis genes. d)Chromosomal location detail view of a chromosome part containing a differential expressed gene. Genes measured on the chip are marked in yellow on the chromosome, differential gene expression is shown above and below the chromosome.

The results of the GO term enrichment analysis are shown in a tree, representing the direct acyclic graph organization of GO. The tree view of the graph is clearer and enables an easier navigation, but leads to multiple entries of GO categories in different branches of the tree. The tree can be searched for GO Identifiers or GO category names. For each node, the number of genes belonging to the category in the subset, in the reference set, the ratio and p-value is shown. An example for the GO term enrichment view is given in figure 4a. We additionally provide a results table for a quick, sortable overview over all categories.

#### Pathway Analysis

The GO term enrichment analysis provides information about the biological process genes are involved in, but does not tell how genes interact. Therefore, another important task in microarray analysis is the identification of regulated pathways. The KEGG PATHWAY [41] database represents networks of molecular interactions and reactions in the cell in a graphical manner. The available pathways provide key information of the functional and metabolic systems within a living cell. We use this database and color differential gene expression of the current working set onto a pathway, allowing the exploration of functional relationships between genes. The enzymatic activity, described by EC numbers [42] in Ensembl, is used for connecting KEGG maps to the probes on the chip. As an enzymatic activity can be catalyzed by more than one gene the pathway view shows different expression values for each different enzyme. If multiple probes exist for one gene, the median value is calculated and used for coloring. Figure 4b shows an example of a KEGG map overlaid with differential expression results. To give a fast overview on which maps are containing what amount of genes for the selected working set, a sortable overview table can be displayed.

For each probe on the microarray all KEGG Maps associated with this probe, if there exist some, can be listed. On the other hand, all probes given on a specific map can be used as probe subset in analysis. All enzymes and genes on a KEGG map can be selected by mouse click, giving detailed information about the corresponding genes in the dataset.

#### Graph View

KEGG pathway information is not available for all genes in a gene subset, because they are not part of a specific pathway, or they are part of a pathway not included in KEGG. However, information about functionally relevant protein interactions is essential for understanding cell behavior. Therefore, an automated display of a STRING summary graph for a subset of genes is implemented in GEPAT. For an easier understanding the differential expression of genes can be mapped onto the graph, giving a fast overview of the expression profile of connected genes. If more than one probe exists for a given gene in the current working subset, the median value is used for visualization. The summary graph can be limited by different types of associations and by the association score provided by STRING.

For each node in the graph, tooltip information is available, and a mouse click on a node provides more information of the selected gene. However, because of the scale-free properties of the gene interaction graph the view is not suitable for larger subsets, as too many nodes do not allow a proper graph layout. An association graph example is shown in figure 4c.

#### Chromosome Location

To investigate the relationship between gene expression changes and physical gene location, a combined view of gene expression and chromosomal location of the probes is available. The mouse cursor can be used to zoom into a specific genomic region. Inside the zoom view, tooltips are provided for each gene, allowing a quick detail investigation at interesting points of the genome, as shown in figure 4d.

## Conclusion

Despite the availability of many programs for microarray data analysis, most of them lack an integration of analysis and interpretation. To understand the effects of differential gene expression an isolated look at genes is not sufficient. It is rather necessary to interpret the results in the context of the cellular network. With the analysis of metabolic or signaling pathways integrating differentially expressed genes, the effects of gene expression on the conditions of cells or tissues can be understood.

GEPAT serves as a toolkit capable of handling the whole progress of microarray data analysis and interpretation in one program. It provides algorithms for the main steps in data analysis, as data import, clustering and differential expression analysis, and offers different methods for data interpretation and visualization, as gene set enrichment analysis or gene association overview. A modular probe and sample selection system allows the usage of analysis and interpretation results as start for new analysis or interpretation methods, facilitating an easy validation of hypotheses or the development of new ones. These integrated capabilities and the build-in annotation support for human microarrays makes GEPAT a powerful tool for microarray data analysis.

It is necessary to be open for new technologies, as biological research develops at fast pace. We implemented large parts of our software in a modular way. Data handling functions serve as a framework that can be extended with various modules for data import, data analysis, data interpretation, subset selection and gene information. As nearly any analysis method can be implemented in this framework, we hope for a future growth of our open-source system. Modules focusing on microRNAs and drug development are currently worked on.

We developed an internet application, focused on easy usage, with a desktop-application like design. This allows a platform-independent remote usage with no need of installation on a local system. With the free availability of the web server, local workgroup installation is possible. To support users untrained in GEPAT, a video tutorial, an online help and test datasets are provided.

## Availability and Requirements

Project Name: GEPAT

Project Home Page: 

Operating Systems: Platform independent, tested on windows and linux

Web browser: tested with Internet Explorer 6 and Mozilla Firefox [43]

Programming language: Java > 1.5, R > 2.2

Other requirements: MySQL 5.0, Apache Tomcat 5.0, JSF 1.1

Licence: Free for academic or commercial users under the GNU Lesser General Public Licence (LGPL)

Example Webserver Home Page: 

## Authors' contributions

MW created the software and web interface and wrote the manuscript. JE created the data analysis R scripts and gave advice in microarray analysis. JS supervised the project and revised the manuscript. All authors read and approved the final version of the manuscript.
